# Impaired orthostatic heart rate recovery is associated with smaller thalamic volume: Results from The Irish Longitudinal Study on Aging (TILDA)

**DOI:** 10.1002/hbm.25022

**Published:** 2020-04-30

**Authors:** Céline De Looze, Wilby Williamson, Rebecca Hirst, John O'Connor, Silvin Knight, Cathal McCrory, Daniel Carey, Rose‐Anne Kenny

**Affiliations:** ^1^ The Irish Longitudinal Study on Ageing, Trinity College, University of Dublin Dublin Ireland; ^2^ Department of Physiology Trinity College Dublin Dublin Ireland; ^3^ Global Brain Health Institute, Trinity College Dublin Dublin Ireland; ^4^ Oxford Cardiovascular Clinical Research Facility Division of Cardiovascular Medicine, Radcliffe Department of Medicine University of Oxford Oxford UK; ^5^ School of Psychology and Institute of Neuroscience, Trinity College Dublin Dublin Ireland

**Keywords:** autonomic function, heart rate recovery, thalamic volume

## Abstract

The thalamus is a central hub of the autonomic network and thalamic volume has been associated with high‐risk phenotypes for sudden cardiac death. Heart rate response to physiological stressors (e.g., standing) and the associated recovery patterns provide reliable indicators of both autonomic function and cardiovascular risk. Here we examine if thalamic volume may be a risk marker for impaired heart rate recovery in response to orthostatic challenge. The Irish Longitudinal Study on Aging involves a nationally representative sample of older individuals aged ≥50 years. Multimodal brain magnetic resonance imaging and orthostatic heart rate recovery were available for a cross‐sectional sample of 430 participants. Multivariable regression and linear mixed‐effects models were adjusted for head size, age, sex, education, body mass index, blood pressure, history of cardiovascular diseases and events, cardiovascular medication, diabetes mellitus, smoking, alcohol intake, timed up‐and‐go (a measure of physical frailty), physical exercise and depression. Smaller thalamic volume was associated with slower heart rate recovery (−1.4 bpm per 1 cm^3^ thalamic volume, 95% CI −2.01 to −0.82; *p* < .001). In multivariable analysis, participants with smaller thalamic volumes had a mean heart rate recovery −2.7 bpm slower than participants with larger thalamic volumes (95% CI −3.89 to −1.61; *p* < .001). Covariates associated with smaller thalamic volume included age, history of diabetes, and heavy alcohol consumption. Thalamic volume may be an indicator of the structural integrity of the central autonomic network. It may be a clinical biomarker for stratification of individuals at risk of autonomic dysfunction, cardiovascular events, and sudden cardiac death.

**Nonstandard Abbreviations and Acronyms**
ΔHR(*t*): difference from baseline value of heart rate at time (*t*) where *t* is time in seconds after standing
BMI: body mass index
BP: mean arterial blood pressure
CVDE: Cardiovascular diseases and event
CVmeds: cardiovascular medications
HR: heart rate
sHR: supine baseline heart rate
HRR: heart rate recovery
HRR10|30s: speed of heart rate recovery between 10 and 30 s after standing = (ΔHR30s − ΔHR10s)
TUG: time up and go

## 1 INTRODUCTION

Heart rate response to physiological stressors and the associated recovery patterns provide reliable indicators of both autonomic function and cardiovascular (CV) risk (McCrory et al., 2016). Orthostatic challenge (standing up) is a physiological stressor which involves baroreflexes, via baroreceptors, blood pressure, and heart rate response. The baroreflex is involved in the regulation of blood pressure, by controlling heart rate and modulating parasympathetic and sympathetic outflow. Seconds after standing, the heart rate increases due to abrupt inhibition of vagal activity via the baroreflex. Peak heart rate is reached at about 10 seconds through a product of vagal inhibition and sympathetic stimulation. Heart rate rapidly decelerates after this, coupled with a stabilization of arterial pressure and vagal reactivation (Borst et al., 1982; Finucane, 2014; Wieling, 1991). The speed of heart rate recovery is a measure of homeostatic flexibility (or adaptive capacity to withstand and respond to stressors) of the CV system, in addition to neuromuscular, vascular and cardiopulmonary baroreflexes, systems which contribute to orthostasis. Attenuated heart rate recovery is associated with increased risk of CV events (McCrory et al., 2016) and with all‐cause mortality (Cole, Blackstone, Pashkow, Snader, & Lauer, 1999). It is attributed to dysfunction of vagal and sympathetic activation.

A growing body of multidisciplinary research describes the central autonomic network and brain‐heart axis co‐ordinating physiological response to orthostatic challenge (Kimmerly, 2017; Tahsili‐Fahadan & Geocadin, 2017). A complex matrix of receptors and neurotransmitters driving anticipatory, inhibitory and stimulatory functions contributes to central autonomic control (Pattinson et al., 2009; Rodenkirch, Liu, Schriver, & Wang, 2019). Several densely connected cortical and subcortical regions known as the “brain rich club” and forming the allostatic interoceptive network, are believed to provide the neural framework which facilitate the integration of task and time dependent functions observed during orthostasis (Honey et al., 2009). These hub regions involved with central baroreflex‐mediated autonomic regulation include the superior parietal, superior frontal, cingulate cortices, and precuneus, basal ganglia, insula, hippocampus, amygdala, thalamus, and cerebellum (Kimmerly, 2017; Van Den Heuvel & Sporns, 2011). Recent experimental studies have highlighted the role of thalamic function in pre‐excitation of motor pathways and anticipation of movement (Gao et al., 2018). The thalamus may be central to maintaining optimal autonomic control and the complex autonomic response to standing.

Tissue volume in the thalamus reflects cortico‐thalamic connectivity and the number of neurons in topographically connected thalamic and cortical regions are closely related (Stevens, 2001). There is emerging evidence suggesting that thalamic atrophy and associated loss of thalamic integrity and functional connectivity may reflect CV function. Acute thalamic injury and chronic neurodegeneration are associated with cardiac dysfunction and impaired CV capacity (Motl et al., 2015). Moreover loss of thalamic volume and reduced functional connectivity are associated with high‐risk phenotypes for sudden cardiac death in epilepsy and schizophrenia (Wandschneider et al., 2015). Slow heart rate recovery, in otherwise health populations, is a risk factor for sudden cardiac death (Jouven et al., 2005). Loss of thalamic structural integrity and functional connectivity may be a contributor to risk patterns associated with slow heart rate recovery.

Emerging evidence suggests that thalamic volume, integrity, and connectivity may be associated with autonomic function. In this study, we identify risk factors related to thalamic atrophy in a large sample of older adults and test the hypothesis that thalamic volume is a predictor of heart rate recovery. In a tertile analysis, we begin by modeling heart rate recovery to standing by tertiles of thalamic volume. We then investigate the association between thalamic volume and heart rate recovery between 10 and 30 s poststanding (i.e., the time period shown to discriminate most highly between highest and lowest thalamic tertiles in our first analysis), to gain insights into the structural correlates of homeostatic flexibility in early stand. Finally, in a linear mixed effect modeling approach, we examine if thalamic volume associations with heart rate recovery are distinct from association with other subcortical basal ganglia, insula, amygdala and hippocampal volumes, as previous work suggests that they may also be involved with central baroreflex‐mediated autonomic regulation (Kimmerly, 2017).

## 2 METHODS

### 2.1 Ethics statement

Ethical approval for the study was obtained from the Faculty of Health Sciences Research Ethics Committee the Trinity College Dublin Research Ethics Committee. Signed informed consent was obtained from all respondents prior to participation. Additional ethics approval was received for the MRI sub‐study from the St James's Hospital/Adelaide and Meath Hospital, Inc. National Children's Hospital, Tallaght (SJH/AMNCH) Research Ethic Committee, Dublin, Ireland. Those attending for MRI were also required to complete an additional MRI‐specific consent form.

### 2.2 Design and participants

We analyzed data from Wave 3 of the Irish Longitudinal Study on Aging (TILDA), a nationally‐representative prospective cohort study of community‐dwelling older adults aged 50 and over, resident in the Republic of Ireland. TILDA's random sampling procedure and study design have been described elsewhere (Donoghue et al., 2018).

### 2.3 MRI protocol and T1w acquisition

Participants aged 65 and over were initially recruited using a random sampling procedure, those aged 50–64 were later targeted (i.e., research nurses informed them of the MR protocol following their health assessment, and screened for MRI contraindications). Participants with pacemakers, cerebral aneurysm clips, or other MRI contraindications were excluded prior to selection. Scanning was completed at the National Centre for Advanced Medical Imaging (CAMI), St. James' Hospital, Dublin, via 3 T Philip's Achieva system and 32‐channel head coil. The protocol included a variety of scans, including a T1‐weighted MR image acquired using a 3D Magnetisation Prepared Rapid Gradient Echo (MP‐RAGE) sequence, with the following parameters: FOV (mm): 240 × 218 × 162; 0.9 mm isotropic resolution; SENSE factor: 2; TR: 6.7 ms; TE: 3.1 ms; flip angle: 9°. The MRI data was obtained with a mean delay of 62 days after the health assessment.

### 2.4 MRI data inspection

All T1w images were analyzed using the FreeSurfer software version 6.0 (Dale, Fischl, & Sereno, 1999). The technical details of FreeSurfer procedures have been described elsewhere. All unprocessed input volumes were inspected for evidence of image artifact and white matter lesions.

### 2.5 Volumetric measures

Right and left thalamic volumes were measured automatically via FreeSurfer pipeline and extracted from FreeSurfer subcortical segmentation statistical output (*aseg*). Right and left volumes were summed for each participant to obtain the total thalamic volume in cubic millimeters. Thalamic volume was then converted to cubic centimeters. Volumetric measures were also extracted for other sub‐cortical areas (the amygdala, hippocampus, caudate, putamen, pallidum, and insula), which are also potential higher centers involved in central autonomic regulation, using the same procedure. Estimated total intracranial volume (eTIV) and overall gray matter volume were also extracted and converted to cubic centimeters.

### 2.6 Active stand protocol

Participants who attended the health center completed a lying‐to‐standing orthostatic test. The active stand protocol employed in TILDA has been described in detail elsewhere (Soraghan et al., 2014). Briefly, participants were asked to lie in a supine position for 10 min. They were then asked to stand as quickly as possible, with or without the assistance of the research nurse, and remained standing for 3 min.

### 2.7 Heart rate measurement

Beat‐to‐beat heart rate (HR) and blood pressure during the active stand test was monitored over 3 min using noninvasive digital photoplethysmography (Finometer, Finapres Medical Systems, Arnhem, the Netherlands; Guelen et al., 2003). HR was downsampled to 1 Hz, and an 11‐s median filter and a 10 second moving average filter was applied for smoothing. The time of standing (or zero time point) for each individual ‐ which corresponds to the moment when the participant lifts their torso off the bed, was estimated using the Finometer height correction unit (O'Connor et al., 2020).

Supine baseline heart rate (sHR) was first extracted and calculated as the mean value between 60 and 30 s prior to standing, during the supine rest period.

HR values at each 10‐s time point after standing were then extracted. These values are denoted HR(*t*), where (*t*) is the time in seconds (s) after standing and takes on values of 10–180 s. Differences from baseline ΔHR(*t*) were generated by subtracting the HR(t) values at each time point from supine baseline heart rate (sHR).

The speed of heart rate recovery between 10 and 30 s after standing (HRR_10|30s_) was also extracted and calculated as the difference in ΔHR(t) values between 10 and 30 s. The smaller the heart rate recovery value, the faster the speed, indicative of a healthy response. At a population level, peak heart rate is reached at about 10s after standing and rapidly declines after this point once blood pressure is stable, which generally occurs within the first 30 s of standing (Borst et al., 1982).

### 2.8 Covariates

Covariates in our analyses (specified in detail below) included age, sex, education, eTIV, mean arterial blood pressure (BP), pre‐existing self‐reported physician‐diagnosed cardiovascular diseases and events (CVDEs), use of cardiovascular medications (CVmeds), diabetes, body mass index (BMI), smoking, alcohol intake, timed up‐and‐go (TUG), physical activity, and self‐reported measure of depression (CESD). Covariates were selected based on previous literature showing the impact of these factors on tissue volumes and/or CV function, representing potential confounders of the relationship between thalamic volume and heart rate response to standing. The eTIV was included to control for individual differences in head size. The TUG measure was included to control for physical frailty (mobility on standing).

Educational attainment corresponds to the highest level of education achieved (none/primary, secondary, or tertiary/higher). Blood pressure was measured using beat‐to‐beat digital plethysmography (Guelen et al., 2003). Mean arterial blood pressure (BP) was derived as the true integrated mean pressure between the current and the next upstroke. Baseline (supine position) was calculated as the mean value between 60 and 30 s prior to standing. Self‐reported doctor‐diagnosed CVDEs included history of angina, heart attack, congestive heart failure, stroke, transient ischemic attack, and atrial fibrillation. Due to the relatively low number of CVDEs in the observed sample, data were pooled to create a dichotomous CVDE measure, for absence (CVDE free) or presence (1+ CVDEs) of CVDEs. A breakdown of the number of CVDEs for the observed sample is given in Supporting Information 3. Self‐reported medication use was cross‐checked with medication labels and assigned WHO Anatomic Therapeutic Chemical (ATC) classification codes. In our analyses, we included five classes of CVmeds: (a) antiadrenergics agents (C02); (b) diuretics (ATC C03); (c) beta‐blockers (ATC C07A); (d) calcium channel blockers (ATC C08C, C08D, and C08E); (e) RAAS blockers, including angiotensin converting enzyme inhibitors (ATC C09A), angiotensin II receptor blockers (ATC C09C) and direct renin inhibitors (ATC C09X). A dichotomous variable was generated to indicate usage of any of these CV medications. Diabetes mellitus was represented as a dichotomous variable for presence or absence of the disease. BMI was calculated by dividing the participants' weight in kilograms by height in meters squared. Participants' height and weight were measured using calibrated measuring equipment. Smoking was categorized using the cross‐classification of smoking status (never, past, and current) and duration (years) to create a five‐level variable: (a) never smoked, (b) past smoker for less than 30 years; (c) past smoker for 30 years and more; (d) current smoker for less than 30 years; (e) current smoker for 30 years and more. The CAGE questionnaire (Mayfield, McLeod, & Hall, 1974) was used as measure of problematic alcohol exposure and is represented as a binary variable. The TUG test (Kenny et al., 2013) required participants to stand up from a chair, walk 3 m and turn around, walk back to the chair and sit down again. The test duration was recorded in seconds. Physical activity was recorded through the International Physical Activity Questionnaire (IPAQ [Hagströmer, Oja, & Sjöström, 2006]; short‐form) and is represented as a three‐level variable: low, moderate, and high. Symptoms of depression were assessed using the Centre for Epidemiological Studies Depression Scale (CESD [Radloff, 1977]) and are represented as a continuous variable.

### 2.9 Statistical analyses

All statistical analyses were performed using the R software version 3.5.2 (R Foundation for Statistical Computing, Vienna, Austria [Team, 2018]).

#### 2.9.1 Data descriptives

The observed sample was first characterized for all participants and per tertile of thalamic volume_._ Continuous variables were described as unadjusted means with standard deviations (*SD*); categorical variables were given as number (*N*) and percentage (%) of the studied sample. For descriptive purposes, separate ordinary least‐square, and logistic regressions (where appropriate) were used to provide an overview of the age, sex, education and head‐size‐adjusted association of each of these variables with thalamic volume tertiles. To estimate selection bias, the observed sample was also compared to the rest of the cohort who attended the center‐based health assessment at Wave 3 but did not undergo an MRI. Separate ordinary least‐square and logistic regressions (where appropriate) were used to assess differences between the two groups.

#### 2.9.2 Analysis 1

Linear mixed‐effects regression analyses were first carried out to explore the association between heart rate response to standing and thalamic volume tertile. Thalamic tertiles were used to estimate critical thalamic volume range whereby volume reduction is associated with higher CV risk as measured per heart rate recovery. The differences in HR from baseline (ΔHR(*t*)) were entered as the dependent variable. Fixed effects included thalamic volume tertile (with the third tertile—larger volumes—as the reference level) and time (*t*) at poststand (10–110 s) with an interaction term. Poststand measures at 120–180 were not included to focus on the speed of heart rate recovery within the first seconds of standing. Participants constituted the random intercept. Our model was adjusted for head size, age, sex, education, BMI, baseline BP, CVDEs, use of CVmeds, diabetes mellitus, smoking, alcohol intake, TUG, physical exercise and depression. Sensitivity analyses were also carried out to account for age and sex differences in volume size (Supporting Information 4 and 5). In a first analysis, a three‐way interaction term was added to the model to assess a potential sex interaction. In a second analysis, age‐ and sex‐adjusted thalamic volume tertiles were calculated using a linear regression approach and then used as fixed effect in the linear mixed effect model.

#### 2.9.3 Analysis 2

Multivariable linear regression analyses were then carried out to examine the association between the speed of heart rate recovery and thalamic volume within the first 30 s poststanding (the time period shown to discriminate most highly between highest and lowest thalamic tertiles), as insights into the structural correlates of homeostatic flexibility in early stand. The speed of heart rate recovery between 10 and 30 s (HRR_10|30s_) was set as the dependent variable and thalamic volume as the primary predictor variable. Our model was adjusted for the 14 covariates described above. A set of sensitivity analyses were further carried out. First, considering the accumulative evidence that autonomic function is lateralized in the human brain (Oppenheimer, Kedem, & Martin, 1996), we assessed whether the association between thalamic volume and heart rate recovery differs for the left and right hemispheres (Supporting Information 6). We also investigated the potential effect of impaired autonomic cardiac function on overall brain structure by testing whether the association between thalamic volume and heart rate recovery holds when controlling for total gray volume and white matter lesions (Supporting Information 7). Finally, providing the associations between thalamic volume and some of the covariates in *Analysis 1*, we further examined the interaction effect of these covariates on the thalamic volume—heart rate recovery association (Supporting Information 8 **)**.

#### 2.9.4 Analysis 3

Linear mixed effects regression analyses were finally carried out to better understand the role of the thalamus in heart rate response to standing, as compared to other subcortical regions, the hippocampus, amygdala, insula, caudate, pallidum and putamen, which may also be central hubs for CV function. To increase statistical power, we examined the associations between HRR_10|30s_ and these subcortical regions in a single model. In this analysis, the volumes of the subcortical regions were entered as the dependent variable. Fixed effects were HRR_10|30s_ and the subcortical regions with an interaction term. The fixed‐effect covariates were the same as per Analyses 1 and 2. Statistical significance was evaluated at a Bonferroni‐corrected *p* < .007 that accounted for multiple comparisons. Our model was adjusted for the 14 covariates as per Analysis 1 and 2.

All our models were checked for assumption of normality of residuals, homoscedasticity, linearity of relationship, absence, or little multicollinearity of predictors and absence of outliers.

## 3 RESULTS

At Wave 3, participants completed a home‐based computer‐assisted personal interview (*N* = 6,613), a self‐completion questionnaire (SCQ), and a physical and cognitive health assessment at a health center or at home (*N* = 5,304). A subset of the respondents who completed CV tests during the center‐based health assessment (*N* = 4,265) were randomly selected to undergo brain MRI (*N* = 578). Eighteen did not complete MRI due to claustrophobia/nervousness (*N* = 14) or MRI contraindication (*N* = 4). Data from one participant with Parkinson's disease and data with evidence of image artifact (*N* = 33) were excluded. Of the 526 participants with adequate volumetric measures, 450 had active stand data available. Sixteen individuals with atrial fibrillation were excluded due to potential beat‐to‐beat heart rate measurement errors. Four were further excluded due to technical problems/deviance from protocol, resulting in a final sample of 430 individuals. Supporting Information 1 presents the flowchart of the study sample.

### 3.1 Characteristics of the observed sample

Table 1 provides descriptive statistics (unadjusted means and standard deviations) for the study sample and per thalamic volume tertile. Mean age was 67.7 (SD = 7.4); 52% of the participants were female and 44% had attained tertiary level of education; 3% reported at least one CVDEs; 34% reported high blood pressure and 39% were on CV medications. Age, sex, education, and head‐size adjusted regression analyses revealed that individuals in the third (larger) thalamic volume tertile were younger than participants in the first (smaller) and second tertiles (B = 9.49; CI_95_ 7.87, 11.10; B = 5.46; CI_95_ 3.95, 6.96; *p* < .001 respectively); age difference was also statistically significant between the first and second thalamic tertiles (B = 4.03; CI_95_ 3.88, 6.84; *p* < .01). The prevalence of women in the third tertile and second tertile groups was lower than in the first tertile group (B = 0.78, CI_95_ 0.06, 1.51; *p* = .03; B = 0.80, *p* = .01; respectively). There was no significant difference in sex between the second and third tertiles. In terms of CV and lifestyle factors associated with thalamic volume, the prevalence of diabetes mellitus and problematic alcohol intake for individuals with larger thalamic volume were lower than for the two other groups (diabetes mellitus: B = 1.82; CI_95_ 0.05, 3.28; *p* = .008; B = 1.76; CI_95_ 0.64, 3.09; *p* = .003; alcohol: B = 1.21; CI_95_ 0.10, 2.37; p = .03; B = 1.36; CI_95_ 0.46, 2.36; *p* = .004). No significant differences in prevalence of diabetes and alcohol were found between the first and second tertiles. There were no significant differences between the thalamic volume‐defined tertile groups (all pairwise comparisons) for the other descriptive variables. The study sample (*N* = 430) is compared to the rest of the cohort at Wave 3 (*N* = 3,689) in Supporting Information 2.

**TABLE 1:** Characteristics of the observed sample for all participants and per tertile of thalamic volume (*N* = 430)

	Study sample (*N* = 430)	First tertile (smaller volume) (I)	Second tertile (II)	Third tertile (larger volume) (III)
*Demographics and cardiovascular risk profile*				
Age, mean (*SD*)	67.7 (7.4)	70.8 (7.3)*	68.1 (7.1) *	64.3 (6.3)
Female, *n* (%)	223 (52)	108 (75)*	69 (48)	46 (32)
Primary education, *n* (%)	84 (19)	30 (21)	23 (16)	31 (22)
Secondary education, *n* (%)	157 (36)	54 (38)	50 (35)	53 (37)
Tertiary education, *n* (%)	189 (44)	60 (42)	70 (49)	59 (41)
BMI, mean (SD) kg/m^2^	27.9 (4.5)	28.1 (4.4)	27.7 (4.8)	28.0 (4.3)
Baseline mean arterial BP, mean (SD), mmHg	101.1 (13.4)	102.8 (14.8)	100.9 (12.9)	99.6 (12.4)
Supine heart rate, mean (SD), bpm	65.23 (9.85)	66.02 (9.82)	64.92 (9.09)	64.75 (10.59)
Cardiovascular diseases (1+), *n* (%)	14 (3)	6 (4)	5 (3)	3 (2)
Cardiovascular medications (hypertensives), *n* (%)	168 (39)	63 (44)	57 (39)	48 (33)
Self‐reported hypertension, *n* (%)	145 (34)	55 (38)	49 (34)	41 (29)
Self‐reported high cholesterol, *n* (%)	154 (36)	60 (42)	52 (35)	42 (29)
Diabetes mellitus, *n* (%)	31 (7)	11 (8)*	16 (11)*	4 (3)
Low physical activity (IPAQ), *n* (%)	139 (32)	61 (42)	42 (29)	36 (25)
Moderate physical activity (IPAQ), *n* (%)	168 (39)	54 (38)	57 (39)	57 (40)
High physical activity (IPAQ), *n* (%)	106 (24)	26 (17)	38 (26)	42 (29)
TUG mean (SD), s	9.09 (1.7)	9.4 2)	9.2 (1.6)	8.7 (1.5)
Never smoked, *n* (%)	216 (50)	74 (51)	72 (50)	70 (48)
Past smoker (less|more than 30 years), *n* (%)	130 (30)|54 (12)	36 (25)|22 (15)	51 (35)|13 (9)	43 (30)|19 (13)
Current smoker (less|more than 30 years), *n* (%)	4 (1)|24 (5)	1 (0)|9 (6)	1 (0)|6 (4)	2 (0)|9 (6)
Alcohol, *n* (%)	41 (9)	13 (9)*	20 (14)*	8 (5)
Depression (CESD), mean (SD)	3.5 (3.4)	4.1 (3.5)	3.3 (3.3)	3.2 (3.3)
*Brain volume profile*				
Thalamic volume, mean (SD)	12.4 (1.2)	11.1 (0.5)*	12.4 (0.3) *	13.8 (0.6)
Gray matter volume, mean (SD)	585.7 (49.1)	546.5 (32)	582.7 (34.9)	628.2 (40.6)

* *p* < .05 indicates significant differences between thalamic tertiles.

### 3.2 Association between thalamic volume tertiles and heart rate response to standing

**FIGURE 1:**
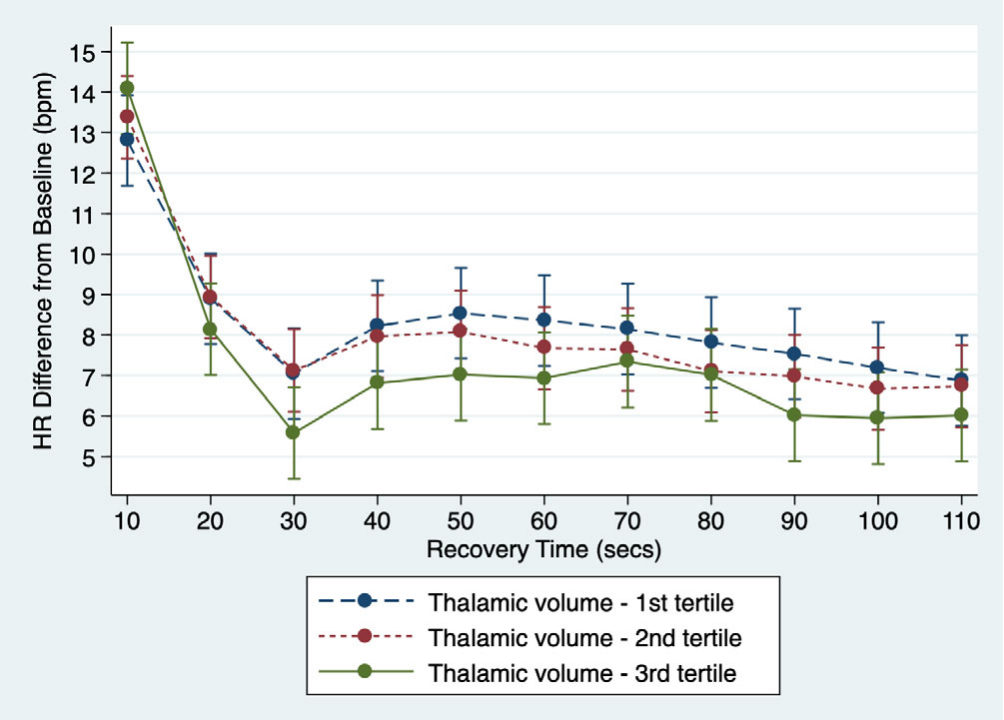
Speed of heart rate recovery after standing by thalamic volume tertile. The hemodynamics of the heart rate response to orthostatic challenge for the first 110 s frame poststanding is presented per thalamic volume tertile. The first tertile represents individuals with smaller thalamic volume; the third tertile, individuals with larger thalamic volume. The estimates of the linear mixed effects model were derived controlling for head size, age, sex, education, BMI, baseline BP, CVDEs, use of AHM, diabetes mellitus, smoking, alcohol intake, TUG, physical exercise and depression. Error bars represent the 95% confidence intervals. As can be seen, individuals with larger thalamic volume (third tertile) are characterized by a steeper initial increase in heart rate and a faster return toward baseline (or steeper drop)

In a preliminary analysis, we first explored the association between heart rate response to standing and thalamic volume tertile, using the larger thalamic volume as the reference level. Figure 1 shows the heart rate response to orthostatic challenge for the two‐min frame poststanding per thalamic volume tertile. Individuals with larger thalamic volume (third tertile) experienced a steeper initial increase in heart rate (higher difference from baseline at 10s after standing) and a faster return toward baseline (or steeper drop) between 10 and 30 s after standing compared to the two other tertiles. Our analyses reveal that difference in heart rate recovery at 30 s was the orthostatic parameter that most clearly differentiated participants with third (larger) vs. second and first (smaller) thalamic volume tertiles, with a difference of 2.26 bpm (CI95 1.12–3.39; *p* < .001) and 2.75 bpm (CI_95_ 1.61–3.89; *p* < .001), respectively. The mean decline in HRR_10|30s_ was −5.75 bpm (CI_95_–6.56 to −4.94; *p* < .001) for the first tertile (smaller thalamic volumes), −6.25 bpm (CI_95−_7.05 to −5.44; *p* < .001) for the second tertile and − 8.51 bpm (CI_95_–9.31 to −7.70; *p* < .001) for the third tertile (larger thalamic volumes) after adjustment for all covariates, meaning that the speed of heart rate recovery is faster for individuals with larger thalamic volume. The results of the sensitivity analyses are presented in Supporting Information 4–5.

### 3.3 Association between thalamic volume and HRR_10__|30s_

Providing that heart rate recovery at 30 s most clearly differentiated thalamic volume tertiles, our second analysis focused on the association between the speed of heart rate recovery and thalamic volume within the first 30 s poststanding. Smaller thalamic volume was associated with slower speed of heart rate recovery between 10 and 30 s after standing. In simple bivariate analysis, HRR_*10|30s*_ was −1.42 beats per minute slower per 1 cm^3^ decrease in thalamic volume (CI_95−_2.01 to −0.82; *p* < .001). The association between thalamic volume and heart recovery remained significant in multivariable modeling (−0.82 bpm slower per 1 cm^3^ thalamic volume, CI_95−_1.49 to −0.13; *p* = .01; Table 2 **)**. The results of the posthoc and sensitivity analyses are presented in Supporting Information 6–8.

**TABLE 2:** Multivariate adjusted association of thalamic volume and covariates with speed of heart rate recovery (HRR_10|30s_) in ordinary least squares regression (*N* = 430)

	Coeff.	[95% CI]
Thalamus (cm^3^)	**−0.82** **	**[−1.49, −0.13]**
*Covariates*		
eTIV	0.00	[0.00, 0.00]
Age (years)	0.16**	[0.04, 0.27]
Female sex	−1.08	[−2.76, 0.60]
*Educational status*		
Primary	REF	‐
Secondary	0.52	[−1.28, 2.33]
Tertiary	0.99	[−0.80, 2.78]
MAP_‐60s|30s_	−0.09***	[−0.14,‐0.00]
Anti‐hypertensive medication	1.23	[−0.15, 2.69]
*Cardiovascular disease status*		
None	REF	‐
One+ CVDEs	−0.88	[−3.54, 1.78]
BMI	−0.27***	[−0.42, −0.12]
Diabetes	0.81	[−1.80, 3.40]
*Physical activity (IPAQ)*		
Low	REF	‐
Medium	−0.17	[−1.70, 1.36]
High	−0.53	[−2.29, 1.23]
Time up and go	0.19	[−0.21, 0.61]
*Smoking history*		
Never smoked	REF	‐
Past smoker <30 years	0.13	[−1.35, 1.61]
Past smoker >30 years	1.14	[−0.91, 3.20]
Current smoker <30 years	5.58*	[−1.20, 12.36]
Current smoker >30 years	3.43*	[0.45, 6.41]
*Alcohol intake (CAGE)*		
Not problematic	REF	‐
Problematic	−0.49	[−2.77, 1.78]
Depression	0.21*	[0.00, 0.40]

*Note*: In this multivariable linear regression analysis, the speed of heart rate recovery between 10 and 30 s (HRR_10|30s_) was set as the dependent variable and thalamic volume as the primary predictor variable. Our model was adjusted for the 14 covariates described below.

Abbreviation: REF, reference category.

*** Significant at the .001 level.

** Significant at the .01 level.

* Significant at the .05 level.

### 3.4 Association between subcortical volumes and HRR_10__|30s_

Our final analysis explored the association between the speed of heart rate recovery within the first 30 s poststanding (HRR_10|30s_) across the volumes of the thalamus, hippocampus, amygdala, insula, caudate, pallidum, and putamen into one single model, to assess their relative contribution to autonomic regulation. Iterative addition of HRR_10|30s_ interaction terms to the fully adjusted model significantly improved the model fit (likelihood ratio test: χ^2^_[5]_ = 31.20, *p* < .001). In the fully adjusted model, the interaction subcortical region × HRR_10|30s_ was significantly different from the interaction thalamus × HRR_10|30s_ for the putamen (B = 0.02; CI_95_ 0.01–0.04; *p* < .001), the caudate (B = 0.03; CI_95_ 0.01–0.04; *p* < .001), the pallidum (B = 0.02; CI_95_ 0.01–0.04; *p* < .001) and the insula (B = 0.03; CI_95_ 0.01–0.04; *p* < .001). The subcortical region × HRR_10|30s_ was not different from the interaction thalamus × HRR_10|30s_ for the amygdala when applying Bonferroni correction (B = 0.01; CI_95_ 0.00–0.03; *p* = .02) and the hippocampus (B = 0.01; CI_95−_0.00–0.02; *p* = .08), suggesting that the amygdala and the hippocampus may serve similar functions.

## 4 DISCUSSION

In this MRI subgroup of a large epidemiological study of aging, smaller thalamic volume was closely related to slower heart rate recovery following orthostatic challenge. The results support a hypothesis for thalamic involvement in the regulation of dynamic autonomic response with potential for increased baroreceptor sensitivity with higher thalamic volume. In the current study, mean heart rate recovery was 2.1–2.7 beats per minute faster in the highest tertile of thalamic volume compared to the other volume tertiles. Age, diabetes and heavy alcohol intake were associated with thalamic volume. Alcohol abuse and diabetes are known to cause autonomic neuropathy and dysfunction (Julian, Glascow, Syeed, & Zis, 2018; Said, 2007). In our study, thalamic volume was 0.56% lower per additional year of age and 3% to 4% lower in association with diagnosis of diabetes and heavy alcohol which is consistent with finding from previous studies (Fjell et al., 2013; Strassburger et al., 1997).

Thalamic atrophy is an established risk indicator of disease burden in epilepsy and multiple sclerosis and a characteristic feature of high‐risk phenotypes for sudden cardiac death (Azevedo et al., 2018; Hilal et al., 2015; Wandschneider et al., 2015). Large epidemiological studies consistently reported slower heart rate recovery after exercise as a predictor of cardiac mortality, which is largely manifested through sudden cardiac death (Cole et al., 1999; Jouven et al., 2005; McCrory et al., 2016). Risk of mortality in association with heart rate recovery has previously been reported from the TILDA study (McCrory et al., 2016). A 1 beat per minute slower heart rate recovery was associated with a 10% increase risk of all‐cause mortality during a mean 4.3‐year follow‐up period (McCrory et al., 2016). The observations in the current study suggest that thalamic integrity helps regulate autonomic control in response to orthostatic challenge.

Maintaining a higher thalamic volume and associated function may be protective against baroreceptor insensitivity. Our findings support the need for future research to understand functional regulation across the heart‐brain axis especially in the context of risk assessment and prevention of sudden cardiac death (Jouven et al., 2005). Thalamic volume may be a valuable biomarker to combine with assessment of heart rate responsiveness and recovery to identify individuals at higher risk of sudden cardiac death.

Recent experimental evidence and imaging studies highlight the importance of thalamic structural integrity and functional connectivity in association with motor, cardiopulmonary, and allostatic interoceptive function (Gao et al., 2018; Honey et al., 2009; Pattinson et al., 2009; Rodenkirch et al., 2019). The finding that thalamic pre‐excitation and activation are primary steps in both motor and sensory responsiveness highlights thalamic function as a potential rate limiting step in the integration of physiological response to orthostatic challenge (Gao et al., 2018). The wider subcortical analysis which identified associations between hippocampal and amygdala volume with heart rate recovery highlights that the thalamus is not an isolated neural hub but that there is a central autonomic network. As described, thalamic nuclei are densely connected with neural hubs within the brain rich club and higher cortical networks. Future work is required to understand temporal dependencies between related neural hubs and the associated autonomic function. Specifically, more understanding is required with regard to potential for neural plasticity in context of aging and CV risk factors. Hippocampal volume is known to decline rapidly with advancing age and targeting hippocampal neuroplasticity is a primary strategy to promote cognitive function and prevent dementia (Firth et al., 2017; Fjell et al., 2013). However, thalamic volume has been identified to decline earlier in the life‐course (Fjell et al., 2013). The expanded understanding of the functional role of the thalamus and the current observations in relation to autonomic function raise the question as to whether promoting and protecting thalamic integrity is a prerequisite for wider neuroplasticity (Bueno‐Junior et al., 2018). Furthermore, thalamic atrophy is a risk factor for cognitive decline and a 1 cm^3^ reduction in thalamic volumes has been associated with twofold increased risk of dementia (Azevedo et al., 2018; Hilal et al., 2015; Wandschneider et al., 2015). With the increased awareness of vascular risk factors as a strong predictors of cerebrovascular changes (Williamson, Lewandowski, Forkert, et al., 2018) and later life dementia (Lourida et al., 2019), there is future merit in investigating heart rate elasticity across the life‐course and association with cognitive function and brain health.

### 4.1 Study limitations

This study has several limitations. First, the MRI sub‐study is cross‐sectional and causality of the observed relationships cannot be inferred. Second, it is possible that the observed associations are explained by undefined, physiological, or pathological pathways, these include factors that influence sensorimotor function and cortical‐thalamic integrity. Experimental studies will be required to explore the interdependency of the current findings. Longitudinal follow‐up would also be required to determine the clinical significance of the observed findings. As such, this study should be considered preliminary and exploratory but does support a need for future work, expanding on other markers of autonomic function such as orthostatic blood pressure and baroreceptor sensitivity.

### 4.2 Strengths

Our study benefits from a unique combination of structural imaging and heart rate response to orthostatic challenge with the ability to control for a large number of potential confounders of the observed associations. The detailed beat‐to‐beat characterization of heart rate response during the first 30 s of active stand also provides a rich data set of dynamic physiological response, which captures the spectrum of autonomic response that may be under central control.

## 5 CONCLUSIONS

Speed of heart rate recovery is a risk factor for CV events and sudden cardiac death. In this study, heart rate recovery following orthostatic challenge is demonstrated to be slower in association with smaller thalamic volume. Thalamic volume may be an indicator of the structural integrity of the central autonomic network. Measurement of thalamic volume could have important applications as a clinical biomarker for risk stratification of individuals at risk of autonomic dysfunction and sudden cardiac death. Future research is required to explore if combined physiological autonomic stress testing with structural imaging across the heart‐brain axis can improve CV risk phenotyping.

## Supporting information


**Appendix** **S1.** Supporting Information.

## Data Availability

Data available on request due to privacy/ethical restrictions
