# Supplementary material for: Impaired orthostatic heart rate recovery is associated with smaller thalamic volume: Results from The Irish Longitudinal Study on Aging (TILDA)
Source: Hum Brain Mapp. 2020 Apr 30;41(12):3370–8. doi: 10.1002/hbm.25022 (PMC7375046; doi:10.1002/hbm.25022)
Supplement: Supplementary file 1 — Appendix S1. Supporting Information. [file HBM-41-3370-s001.docx]

**Supporting information**

**Supporting information 1**

**Figure S1:** Flowchart of the study sample.

Wave 3 participants aged >= 50 years

N=6,613

Home base health assessment (n=1,072)

Did not attend health assess. (n=1,276)

N=2,348

MRI data with motion artefact (n=33) + Parkinson’s disease (n=1)

N=34

Individuals with atrial fibrillation (N=16) and active stand data with technical errors (N=4) N=20

Good quality MRI data with active stand data available for analysis

N=450

No active stand data

N = 76

Good quality MRI data available for analysis

N=526

T1w datasets acquired

N=560

No data obtained due to claustrophobia/nervousness (n=14) or MRI contraindication (n=4)

N=18

MRI sub-study

N=578

Eligible for analysis of thalamic volume and active stand beat-to-beat heart rate measures

**N=430**

Centre-based health assessment

N=4,265

Did not undergo brain MRI

N=3,687

**Supporting information 2.** The table below provides descriptive statistics for the rest of the cohort at Wave 3 in comparison with the study sample for information purposes. Separate ordinary least-square and logistic regressions (where appropriate) were used to assess differences between the two groups.

**Table S1.** Characteristics of the study sample (N=430) and the rest of the cohort who attended the centre-based health assessment at Wave 3 but did not undergo an MRI or were further excluded from MRI analysis (N=3,689). * (p<.05) indicates significant differences between the study sample and the rest of the cohort.

|  | **Study Sample**  **(N=430)** | **Rest of the cohort (N=3,689)** |
| --- | --- | --- |
| **Demographics & cardiovascular risk profile** | | |
| Age, mean (sd) | 67.75 (7.42) | 64.43 (8.15)* |
| Female, n (%) | 223 (52) | 2053 (56) |
| Primary education, n (%) | 84 (20) | 634 (17) |
| Secondary education, n (%) | 157 (36) | 1505 (41) |
| Tertiary education, n (%) | 189 (44) | 1549 (42) |
| BMI, mean (sd) kg/m2 | 27.96 (4.52) | 28.55 (5.15)* |
| Baseline mean arterial BP, mean (sd), mmHg | 101.16 (13.45) | 100.40 (13.41) |
| Cardiovascular diseases (1+), n (%) | 14 (7) | 312 (8)* |
| Cardiovascular medications (hypertensives), n (%) | 168 (40) | 1424 (38) |
| Self-reported hypertension, n (%) | 145 (34) | 1210 (33) |
| Self-reported high cholesterol, n (%) | 154 (35) | 1375 (37) |
| Diabetes mellitus, n (%) | 31 (7) | 257 (7) |
| Low physical activity (IPAQ), n (%) | 139 (32) | 1224 (33) |
| Moderate physical activity (IPAQ), n (%) | 168 (38) | 1325 (36) |
| High physical activity (IPAQ), n (%) | 106 (24) | 950 (26) |
| TUG mean (sd),sec | 9.09 (1.73) | 9.15 (2.33) |
| Never smoked, n (%) | 216 (50) | 1697 (46) |
| Past smoker (less \| more than 30 years), n (%) | 130 (30) \|  54 (13) | 1129 (30) \|  458 (12) |
| Current smoker (less \| more than 30 years), n (%) | 4 (1) \|  24 (5) | 43 (1) \|  349 (9) |
| Alcohol, n (%) | 41 (10) | 459 (12) |
| Depression (CESD), mean (sd) | 3.54 (3.42) | 3.91 (3.75)* |

**Supporting information 3.** Table S2 below provides a breakdown of the number of doctor-diagnosed self-reported cardiovascular diseases and events in the study sample for information purposes.

**Table S2.** Number (N) of doctor-diagnosed self-reported cardiovascular diseases and events in the study sample.

| **Cardiovascular diseases/ events** | **N (%)** |
| --- | --- |
| Angina | 7 (2) |
| Heart attack | 2 (0.5) |
| Heart fail | 1 (0.5) |
| Stroke | 2 (0.5) |
| Transient ischemic attack | 3 (1) |

**Supporting information 4.** Sensitivity analyses were carried out to account for sex differences in volume size. In a linear mixed effects model, differences in HR from baseline (ΔHR(t)) were entered as the dependent variable. Fixed effects included thalamic volume tertile (with the third tertile – larger volumes – as the reference level), time (t) at post-stand (10 to 110 s) and sex, with an interaction term (three-way interaction). Participants constituted the random intercept. Our model was adjusted for head size, age, education, BMI, baseline BP, CVDEs, use of CVmeds, diabetes mellitus, smoking, alcohol intake, TUG, physical exercise and depression. The interaction between thalamic tertile, time and sex was not significant (p>0.05).

**Supporting information 5.** Sensitivity analyses were carried out to account for age and sex differences in volume size. Age- and sex-adjusted thalamic volume tertiles were calculated using a linear regression approach. In a linear mixed effects model, differences in HR from baseline (ΔHR(t)) were entered as the dependent variable. Fixed effects included age- and sex-adjusted thalamic volume tertile (with the third tertile – larger volumes – as the reference level) and time (t) at post-stand (10 to 110 s), with an interaction term. Participants constituted the random intercept. Our model was adjusted for head size, age, sex, education, BMI, baseline BP, CVDEs, use of CVmeds, diabetes mellitus, smoking, alcohol intake, TUG, physical exercise and depression.

Individuals with larger thalamic volume (third tertile) experienced a steeper initial increase in heart rate (higher difference from baseline at 10s after standing) and a faster return toward baseline (or steeper drop) between 10 and 30 s after standing compared to the third tertile. Our analyses reveal that difference in heart rate recovery at 30 s was the orthostatic parameter that most clearly differentiated participants with third (larger) vs. first (smaller) thalamic volume tertiles, with a difference of 1.65 bpm (CI_95_ 0.51,2.80; p=0.005). The mean decline in HRR_10|30s_ was of -6.01 bpm (CI_95_ -6.82 to -5.20; p<.001) for the first tertile (smaller thalamic volumes), -6.82 bpm (CI_95_  -7.63 to -6.02; p<.001) for the second tertile and -7.67 bpm (CI_95_ -8.48 to -6.86; p<.001) for the third tertile (larger thalamic volumes) after adjustment for all covariates.

**
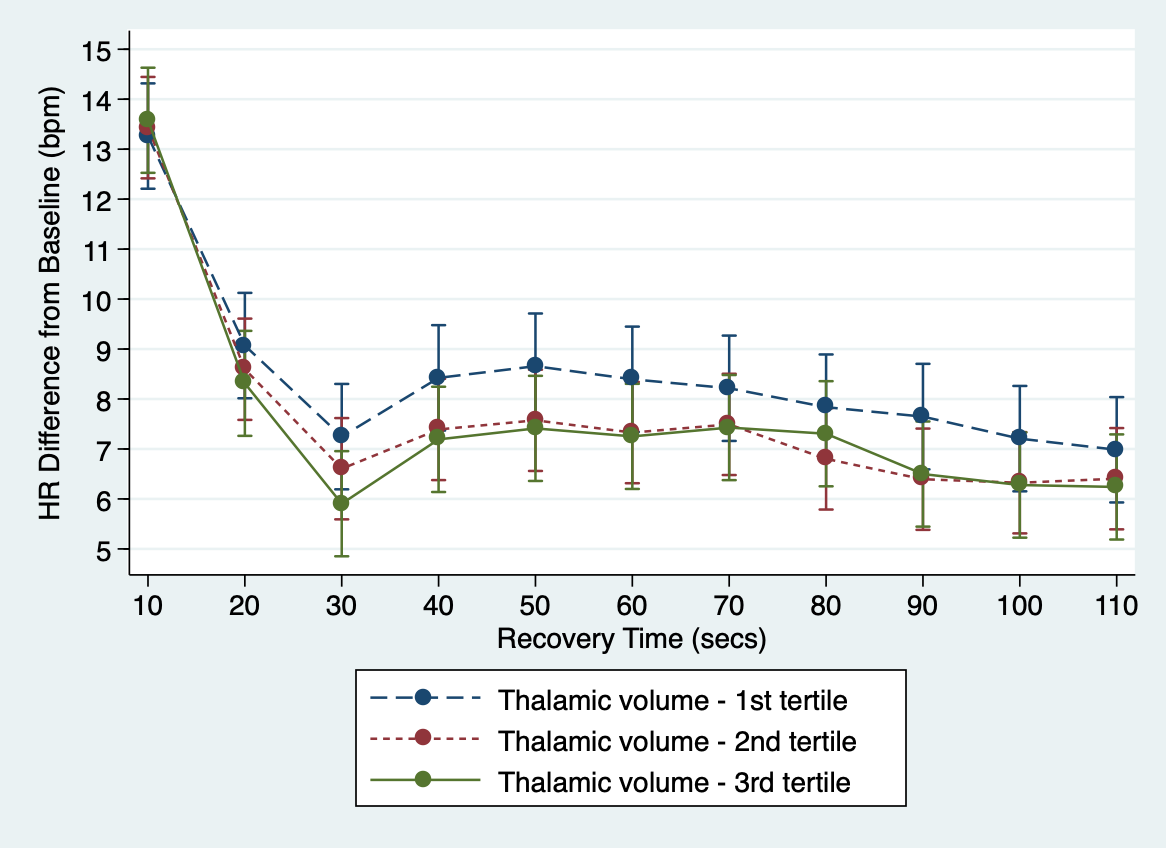
Figure S1.** Speed of heart rate recovery after standing by age- and sex-adjusted thalamic volume tertile. The estimates of the linear mixed effects model were further derived controlling for head size, age, sex, education, BMI, baseline BP, CVDEs, use of AHM, diabetes mellitus, smoking, alcohol intake, TUG, physical exercise and depression. Error bars represent the 95% confidence intervals.

**Supporting information 6:** Considering the accumulative evidence that autonomic function is lateralized in the human brain (Oppenheimer, 1992), post-hoc analyses were carried out to assess whether the association between thalamic volume and heart rate recovery differs for the left and right hemispheres. Results show that both the left and right thalamic volumes when taken separately are associated with heart rate recovery.

**Table S3**: Multivariate Adjusted Association of ***left*** thalamic volume and covariates with Speed of Heart Rate Recovery (HRR_10|30s_) in Ordinary Least Squares Regression (N=430)

|  | *Coef.* | *[95% CI]* |
| --- | --- | --- |
| **Left thalamus (cm^3^)** | **-1.61*** | **[-2.90, -0.32]** |
|  |  |  |
| **Covariates** |  |  |
| eTIV | 0.00 | [0.00, 0.00] |
| Age (years) | 0.16** | [0.04, 0.27] |
| Female sex | -1.13 | [-2.81, 0.54] |
|  |  |  |
| *Educational Status* |  |  |
| Primary | REF | - |
| Secondary | 0.53 | [-1.27, 2.34] |
| Tertiary | 1.01 | [-0.77, 2.80] |
|  |  |  |
| MAP_-60s\|30s_ | -0.09*** | [-0.14,-0.00] |
| Anti-hypertensive medication | 1.28 | [-0.13, 2.70] |
|  |  |  |
| *Cardiovascular Disease Status* |  |  |
| None | REF | - |
| One+ CVDEs | -0.89 | [-3.55, 1.77] |
|  |  |  |
| BMI | -0.27*** | [-0.41, -0.12] |
|  |  |  |
| Diabetes | 0.87 | [-1.71, 3.46] |
|  |  |  |
| *Physical activity (IPAQ)* |  |  |
| Low | REF | - |
| Medium | -0.18 | [-1.72, 1.34] |
| High | -0.57 | [-2.33, 1.19] |
|  |  |  |
| Time-Up&Go | 0.21 | [-0.19, 0.62] |
|  |  |  |
| *Smoking History* |  |  |
| Never smoked | REF | - |
| Past smoker <30 years | 0.01 | [-1.32, 1.64] |
| Past smoker>30 years | 1.19 | [-0.87, 3.25] |
| Current smoker<30 years | 5.48 | [-1.29, 12.26] |
| Current smoker>30 years | 3.52* | [0.55, 6.49] |
|  |  |  |
| *Alcohol intake (CAGE)* |  |  |
| Not problematic | REF | - |
| Problematic | -0.53 | [-2.80, 1.75] |
|  |  |  |
| Depression | 0.21* | [0.00, 0.41] |

*** Significant at the 0.001 level; ** significant at the 0.01 level; * significant at the 0.05 level; REF = reference category

**Table S4**: Multivariate Adjusted Association of ***right*** thalamic volume and covariates with Speed of Heart Rate Recovery (HRR_10|30s_) in Ordinary Least Squares Regression (N=430)

|  | *Coef.* | *[95% CI]* |
| --- | --- | --- |
| **Right thalamus (cm^3^)** | **-1.34*** | **[-2.65, -0.33]** |
|  |  |  |
| **Covariates** |  |  |
| eTIV | 0.00 | [0.00, 0.00] |
| Age (years) | 0.17** | [0.06, 0.28] |
| Female sex | -0.99 | [-2.67, 0.68] |
|  |  |  |
| *Educational Status* |  |  |
| Primary | REF | - |
| Secondary | 0.53 | [-1.28, 2.34] |
| Tertiary | 0.99 | [-0.79, 2.79] |
|  |  |  |
| MAP_-60s\|30s_ | -0.09*** | [-0.14,-0.00] |
| Anti-hypertensive medication | 1.26 | [-0.17, 2.68] |
|  |  |  |
| *Cardiovascular Disease Status* |  |  |
| None | REF | - |
| One+ CVDEs | -0.82 | [-3.50, 1.84] |
|  |  |  |
| BMI | -0.27*** | [-0.41, -0.12] |
|  |  |  |
| Diabetes | 0.82 | [-1.79, 3.43] |
|  |  |  |
| *Physical activity (IPAQ)* |  |  |
| Low | REF | - |
| Medium | -0.17 | [-1.71, 1.36] |
| High | -0.52 | [-2.28, 1.25] |
|  |  |  |
| Time-Up&Go | 0.18 | [-0.23, 0.59] |
|  |  |  |
| *Smoking History* |  |  |
| Never smoked | REF | - |
| Past smoker <30 years | 0.01 | [-1.35, 1.1] |
| Past smoker>30 years | 1.11 | [-0.95, 3.17] |
| Current smoker<30 years | 5.73 | [-1.05, 12.51] |
| Current smoker>30 years | 3.41* | [0.42, 6.40] |
|  |  |  |
| *Alcohol intake (CAGE)* |  |  |
| Not problematic | REF | - |
| Problematic | -0.41 | [-2.69, 1.86] |
|  |  |  |
| Depression | 0.20* | [-0.00, 0.39] |

*** Significant at the 0.001 level; ** significant at the 0.01 level; * significant at the 0.05 level; REF = reference category.

**Supporting information 7.** Sensitivity analyses were carried out to assess the potential effect of impaired autonomic cardiac function on overall brain structure. We investigated whether the association between thalamic volume and heart rate recovery holds when controlling for Total Grey Volume and White Matter lesions too. Data for 13 participants revealed one or more lesions present. No significant difference in the results was found.

**Table S5**: Multivariate Adjusted Association of thalamic volume and covariates with Speed of Heart Rate Recovery (HRR_10|30s_) in Ordinary Least Squares Regression (N=430).

|  | *Coef.* | *[95% CI]* |
| --- | --- | --- |
| **Thalamus (cm^3^)** | **-0.08*** | **[-1.59, -0.09]** |
| **Covariates**  eTIV | 0.00 | [-0.00, 0.00] |
| Total Grey Volume | 0.00 | [-0.00, 0.00] |
| Lesions | 1.48 | [-2.37, 5.34] |
| Age (years) | 0.16** | [0.03, 0.27] |
| Female sex | -1.11 | [-2.81, 0.64] |
|  |  |  |
| *Educational Status* |  |  |
| Primary | REF | - |
| Secondary | 0.58 | [-1.23, 2.40] |
| Tertiary | 1.05 | [-0.74, 2.86] |
|  |  |  |
| MAP_-60s\|30s_ | -0.09*** | [-0.14,-0.00] |
| Anti-hypertensive medication | 1.23 | [-0.19, 2.66] |
|  |  |  |
| *Cardiovascular Disease Status* |  |  |
| None | REF | - |
| One+ CVDEs | -0.79 | [-3.47, 1.88] |
|  |  |  |
| BMI | -0.27*** | [-0.42, -0.12] |
|  |  |  |
| Diabetes | 0.89 | [-1.72, 3.50] |
|  |  |  |
| *Physical activity (IPAQ)* |  |  |
| Low | REF | - |
| Medium | -0.16 | [-1.70, 1.37] |
| High | -0.58 | [-2.36, 1.19] |
|  |  |  |
| Time-Up&Go | 0.19 | [-0.22, 0.60] |
|  |  |  |
| *Smoking History* |  |  |
| Never smoked | REF | - |
| Past smoker <30 years | 0.15 | [-1.33, 1.65] |
| Past smoker>30 years | 1.19 | [-0.90, 3.28] |
| Current smoker<30 years | 5.65 | [-1.14, 12.44] |
| Current smoker>30 years | 3.40* | [0.42, 6.39] |
|  |  |  |
| *Alcohol intake (CAGE)* |  |  |
| Not problematic | REF | - |
| Problematic | -0.52 | [-2.81, 1.76] |
|  |  |  |
| Depression | 0.20* | [-0.00, 0.40] |

*** Significant at the 0.001 level; ** significant at the 0.01 level; * significant at the 0.05 level; REF = reference category.

**Supporting information 8.** Providing the association between thalamic volume and diabetes and alcohol in the descriptive analyses, post-hoc analyses were carried out to investigate the interaction effect of diabetes and alcohol on the thalamic volume – heart rate recovery association. The speed of heart rate recovery between 10 and 30 seconds (HRR_10|30s_) was set as the dependent variable and thalamic volume as the primary predictor variable in the multivariable linear regression analyses. Diabetes and alcohol were added as interaction terms in separate analyses. Our models were adjusted for the fourteen covariates described in the manuscript. The interaction with diabetes (p=0.73) and alcohol (p=0.65) were not significant.
